# Evaluating the Implementation and Delivery of a Social Prescribing Intervention: A Research Protocol

**DOI:** 10.5334/ijic.3087

**Published:** 2018-03-21

**Authors:** Julia Vera Pescheny, Yannis Pappas, Gurch Randhawa

**Affiliations:** 1 Institute for Health Research, University of Bedfordshire, Luton, UK

**Keywords:** social prescription, implementation, delivery, patient engagement, integrated care

## Abstract

**Background:**

In response to the increasing numbers of people with (multiple) chronic conditions, the need for integrated care is increasing too. Social prescribing is a new approach that aims to integrate the social and healthcare sector to improve the quality of care and user experience. Understanding main stakeholders’ perceptions and experiences is key to the implementation of social prescription and for informing future initiatives.

**Objectives:**

This paper presents the protocol of a qualitative research study to explore factors that (i) facilitate and hinder the implementation of a social prescribing pilot in the East of England, and (ii) affect the uptake, adherence, and completion rates by service users.

**Methods:**

A qualitative study including semi-structured interviews with managers, health professionals, service providers, navigators, and service users. Iterative thematic analysis will be used to analyse the data.

**Conclusion:**

This study will produce evidence on factors that hinder and facilitate the implementation of a social prescribing programme, as well as factors affecting the engagement, and non-engagement, of service users. Findings can contribute to the development of an evidence base for social prescription programmes in the UK, and inform practice, policy, and future research in the field.

## Introduction

In England, more than 15 million people have a Long Term Condition (LTC) [1]. The numbers of people with chronic conditions are growing, with a particularly rapid rise in the number of people with three or more chronic conditions at once [1]. When it comes to manage chronic disease in the English National Health System (NHS), service delivery tended to fragment care, both within the sectors of the health service, i.e. primary, secondary, and tertiary care, and between sectors, i.e. health and social care [2]. To meet the complex needs of people with (multiple) chronic conditions, the development of delivery systems that integrate a range of professionals and skills from the cure and care sector are required [3,4]. Integrated care is expected to improve patient safety, quality of care, user experience, and cost-effectiveness through better coordinated and more continuous care [2,5].

There is no single model of integrated care, as there are different types of integration focusing on different patient groups. Integration may focus for example on the three sectors of the health service (vertical integration), the social and health sector (horizontal integration), and the integration of care within one sector [2,5]. This paper will focus on the integration of the social and health sector to enhance quality of care and user experience.

In response to the need of a more integrated delivery system, there were several attempts to introduce the integration of the health and social sector in the English NHS, including social prescription interventions [2,6,7]. Social prescribing is a way of linking patients in primary care with sources of non-medical support, typically provided by the third sector (including charities, voluntary, and community groups) [8]. The interest in social prescribing is rising because of the increasing burden of LTCs, the cost implications this poses for service delivery, and the crisis in general practice [9,10,11]. Recent studies on social prescribing indicate that there is a potential for health and wellbeing improvements in people with LTCs and reductions in health resource use [10,12,13,14,15]. Despite the need for more robustly evaluated examples in real-world conditions to proceed the integrated care agenda, most evaluations of social prescribing interventions appear to be small scale and limited by poor design and reporting [11,15,16].

This paper describes the design of a qualitative study looking at several outcomes: Factors that hinder and facilitate the implementation of social prescribing interventions as well as factors that affect service user adherence, uptake, and completion. Previous research identified a standardised training programme for navigators, networking events to share best practice [17], and shared understanding of an intervention [18] as facilitators to the implementation of social prescribing interventions. Lack of partnership and service level agreements [18], high staff turnover [19], and low practice staff engagement, including low referral rates form health professionals [18] were identified as barriers to social prescribing interventions. Factors that affect service users’ uptake, adherence, and completion of the programme could be related to service users’ personality traits, health-believes, and motivation [20]. These outcomes are fairly universal in evaluating interventions of integrated care, such as social prescription, and therefore are deemed to be highly generalizable [20,21,22,23]. This study aims to contribute to the development of an evidence base for social prescription programmes in the UK, to inform practice and policy, and to provide insights and learning for the implementation of future social prescription programmes.

### The intervention

Social prescribing is an approach that can bridge the existing gap between the health and third sector [7]. There is no single and agreed understanding of what constitutes social prescribing, hence interventions vary across the United Kingdome in terms of their referral routes, target groups, specific objectives, and range of non-medical support options [16,24]. However, based on previous research in England, social prescribing interventions were delineated into four groups: Signposting, Light, Medium, and Holistic [9].

The research focuses on a holistic social prescribing pilot programme that was implemented in one Clinical Commissioning Group (CCG) area in the East of England (Luton) in 2015. The aims of the pilot are to enhance primary care patient outcomes and the collaboration between community, public, and health services. The social prescribing model is based in primary care and involves navigators who are based in general practices across the four clusters of Luton. In the Luton model, general practitioners refer primary care patients with (i) diabetes and pre-diabetes, (ii) mild to moderate mental health issues, (iii) Chronic Obstructive Pulmonary Disease (COPD), and (iv) carers, with non-medical needs to a navigator. Recognising that people’s health is also determined by social, economic, and environmental factors, the navigators’ role is to assess the non-medical needs of patients. In a next step, the navigators refer, or signpost, patients to sources of support within the third sector, with the aim to improve their health and wellbeing. In order to receive referrals from the social prescription programme, service providers have to complete an accreditation process. Examples of services and activities include art therapy, walking and reading groups, exercise classes, nature-based activities, and volunteering, as well as support with employment, debt, housing, and legal advice. Navigators can refer patients to a maximum of twelve sessions, which are free of charge for service users. Whilst on the social prescription programme, there are no limits to the number of times a service user can meet with a navigator. Therefore, the numbers of sessions the service user can have with a navigator depend on the service user’s needs.

### Philosophical perspective

This research is based on the critical realist paradigm. Critical realism is a relatively contemporary philosophy of science that offers an alternative to the established purist paradigms of positivism and interpretivism [25,26]. Critical realism was founded by Roy Bhaskar in the 1970s and 1980s, and it was further developed by Margaret Archer, Mervyn Hartwig, Tony Lawson, Alan Norrie and Andrew Sayer [27]. Although it is a relatively new philosophical stance in health research, it is becoming increasingly influential [28].

The ontological assumptions (i.e. the nature of reality) of critical realism is that the world is a stratified, open system [29,30,31]. Critical realism assumes an inter-related stratified ontology divided into three domains: the real (those structures and mechanisms that generate phenomena), the actual (those aspects of reality that occur but may not be observable), and the empirical (those aspects of reality that can be directly or indirectly experienced and observed) [28,32].

Bhaskar draws a distinction between reality and the accepted knowledge of that reality [33]. Although reality is believed to be independent of knowledge and perception (intransitive domain), the generation of knowledge about that reality is socially derived and a human activity that is dependent on theories, methods, models, and techniques used by researchers at a certain time and place (transitive domain) [32,34]. Hence, critical realists belief that knowledge is articulated in two dimensions:

“It is a socially produced knowledge of a neutral (human independent) thing” [21, p. 65]

The distinction between the intransitive and transitive domains points out that despite ontological realism, epistemological (i.e. our knowledge of reality) relativism is adopted [35]. Therefore, critical realists view the process of scientific knowledge construction as socially constructed, historically emergent, political, and imperfect [36]. Contrary to positivists, critical realists do not restrict reality to structures, processes, and mechanisms that are visible and empirically observable.

Critical realists believe that observable phenomena are embedded in a wide range of mechanisms, processes, and structures which occur under certain conditions, and therefore consider the importance of contextual factors (including structural, organizational, and human constrains) in knowledge generation [32]. Their main claim is that the implementation of an intervention, as well as service users’ behaviour and decision-making, are influenced by contextual factors [37]. This has been an important guiding principle in the research described in this paper, as the researchers aimed to embed the research findings in the wider research context. Thus, critical realism provides a strong philosophical ground for this qualitative research study.

### Research aims

The first aim of this study is to identify factors that hinder and facilitate the implementation of the Luton social prescribing pilot. The second aim is to explore factors that affect service user adherence, uptake, and completion of this social prescribing programme.

## Research methods

### Study design

A qualitative study design will be employed to address the two objectives of the research. In order to develop a comprehensive understanding of factors that hinder and facilitate the implementation of the Luton social prescribing programme, the perspectives of four stakeholder groups, namely health professionals, navigators, managers, and service providers from the third sector, are studied for the first objective. Managers include all stakeholders that are involved in the implementation process, for example, local CCG members, steering group members, the programme managers, and public health consultants. To gain an understanding of the factors that affected the engagement, and non-engagement, of service users, the perspective of service users (including those who didn’t engage with the programme after being referred by a GP) are studied. This research method enables the researchers to access the stakeholders’ insights into the details of the implementation process and engagement of referred primary care patients. This study is part of a PhD study, which takes three years.

To protect the participants’ safety, rights, wellbeing and dignity, this research has been reviewed and given favourable opinion by the Institute for Health Research Ethics Committee at the University of Bedfordshire, National Research Ethics Committee (REC), and the National Health Research Authority (HRA).

### Setting

The research described in this protocol will take place in the East of England in Luton.

### Sample

Purposive sampling is a method of non-probability sampling, in which participants are selected based on certain predefined criteria, such as experiences or knowledge [38]. Purposive sampling is often used when small samples are studied using focused data collection methods such as semi-structured interviews [39]. A purposive sampling strategy will be employed to maximise the representativeness and diversity of stakeholders involved in the social prescription programme. Hence, a purposive sampling strategy will be adopted to select health professionals, managers, navigators, and service providers involved in the implementation of the pilot, as well as service users across different engagement levels. Based on the number of stakeholders involved in the implementation of the social prescription pilot, it is planned to recruit between 22 and 25 stakeholders (GPs = 4 (one from each participating surgery), navigators = 4 (total number of employed navigators), service providers = 5–8 (of different: size, commissioning status, and services) managers = 9 (based on the number of managers in the pilot) to address the first objective of the study.

To address the second objective, it is planned to recruit between 15 and 20 service users, i.e. primary care patients who were referred to the social prescription programme, across different engagement levels. The inclusion and exclusion criteria for all service users, including those who didn’t engage after the referral to the programme, are displayed in Table 1.

**Table 1:** Inclusion and exclusion criteria for the service user sample.

Inclusion criteria	Exclusion criteria

· Referred to the SP service by a GP · Referred to SP some time over the past year (from the point on when recruitment of participants start in the study) · Sufficient English speaking skills to take part in the study	· Service users with significant hearing impairments

### Recruitment

#### Managers and navigators

Managers and navigators will be invited to take part in the study via email. The email will contain: an invitation letter, an information sheet, and a contact sheet on which potential participants can indicate whether they wish to take part in the study.

### General practitioners

Navigators, the project manager, and members of the CCG agreed to help with the recruitment of general practitioners for the study. They were asked because they have established relationships with involved GPs, which could increase the response rates to the invitation emails. Navigators, the project manager, and members of the CCG will inform GPs about the study through various ways, for example during routine meetings or via email. An invitation to the study, the information sheet, and a contact form will be send out to all involved GPs via email.

### Service providers in the third sector

The project manager and the navigators agreed to help with the recruitment of service providers from the third sector, as they have established relationships with service providers and communicate with them on a regular basis. They will explain the research study to selected service providers and ask them whether they are interested in participating in the study. If so, prospective participants will receive an invitation email, the information sheet, and contact form via the provided email address. To avoid that service providers feel pressured to take part in the study, managers and navigators will highlight that participation in the study is voluntary.

### Service users

To protect the privacy of service users, navigators were asked to help with service user recruitment for this study. Navigators will hand out or send out by post a recruitment pack to eligible service users, consisting of an invitation letter, information sheets, a contact form, and a pre-paid envelope to return the contact form to the researchers. To support navigators and to ensure that consistent information is provided to eligible service users, navigators are provided with a guide for service user recruitment. Additionally, to avoid that eligible service users feel coerced into participation, navigators will highlight that participation is voluntary, confidential, and that taking part will not affect the care patients receive, when handing out the recruitment pack.

### Interview schedules

Given that each stakeholder group has different roles and responsibilities in the implementation process of the social prescription programme, four different interview schedules are developed to explore facilitators and barriers to the implementation. To explore factors affecting the uptake, adherence, and completion of the social prescription programme, a fifth interview schedule is developed for service users. It is planned to conduct all interviews face-to-face. The interviews will last between 30 and 45 minutes.

The interview guides are drafted based on knowledge gained from a literature review and the pathway of the social prescription programme in Luton. Interview schedules are discussed with experts and researchers in the field, as well as piloted with a convenient sample of one or two stakeholders per stakeholder group. The questions in the interview schedules for health professionals, navigators, and service providers are structured around the roles and relevant pathways for each stakeholder group. Hence, questions to health professionals are structured around setting up the programme in a practice, the first part of the social prescription pathway, i.e. the identification and referral of primary care patients, and their reasons for engagement and disengagement. Questions for navigators are structured around their experience of performing the navigator role and establishing themselves in a surgery. Questions for service providers are structured around their reasons for engagement, the accreditation process, and final path of the social prescription programme, i.e. receiving referrals and providing services. As managers are not directly involved in delivering the service, questions for managers are structured around the implementation process in general. By asking a general question, for example *‘What facilitated the implementation process?’*, managers have the opportunity to talk about factors that are relevant from their perspective, determined by their role and responsibilities. In general, all four interview schedules are deigned to support the development of an understanding of facilitators and barriers to the implementation of the social prescription programme.

Similar to the interview schedules for health professionals, navigators, and service providers, the interview questions for service users are structured around the social prescription pathway from a service user’s perspective. This provides the opportunity to explore the reasons for a service user’s behaviour or decisions, by asking, for example, *‘Why did you agree to be referred to the social prescription programme?’* or *‘Why did you attend the services/activities as agreed with the navigator? Why not?’* Going through each stage of the social prescription pathway from a service user’s perspective, supports the development of an understanding of factors affecting the uptake, adherence, and completion of the programme.

### Data collection and storage

Given the context and the aim of the study, stakeholders involved in the implementation and delivery of the social prescribing pilot work together on a daily-basis. Thus, the existing power imbalances between potential participants, i.e. managers and employees, may limit disclosure during group discussion, for example in the form of focus groups [40]. Similarly, it is likely that service users would not feel comfortable discussing their experiences, and reasons for engaging, or not engaging with the programme, in a group [41]. Therefore, semi-structured interviews are the most appropriate data collection tool to explore the insights’ of stakeholders in details while offering a private and confidential environment.

All interviews will be recorded with an audio-recorder, transferred to a password protected work computer, and then transcribed verbatim as a Microsoft Word document. The audio-recordings and Microsoft Word documents will be stored at a password-protected work computer, in a password-protected file.

### Consent

Written consent for the participation in the study, that the interview is audio-recorded, and the use of verbatim anonymised quotes in the PhD thesis, research reports, and articles will be obtained from each participant immediately before the interview.

### Data analysis

Each transcript will be compared to the original audio-recordings for accuracy. To protect the privacy of participants, identifiable information, such as participants’ and their organisations’ names, are removed from the transcripts. Iterative thematic analysis will be used to analyse the data. As described by Braun and Clarke (2006) [42], this study follows the following phases of iterative thematic analysis:

Phase 1: Familiarisation with the data.
Phase 2: Generating initial codes.
Phase 3: Searching for themes.
Phase 4: Reviewing themes.
Phase 5: Defining and naming themes.
Phase 6: Producing the report.

One researcher will conduct the coding and data analysis, under the supervision of two experienced qualitative researchers. The created codes will be developed into categories and themes, and reviewed and refined throughout the process. The computer assisted qualitative data analysis programme QSR NVivo 11 will be used to assist the analysis. Following the critical realist philosophy, contextual factors and their influences on participants’ experiences, behaviour, and decisions will be considered in the analysis. Hence, the experiences and views of participants will be related to social structures, networks, and other contextual factors. This is in line with critical realist research that aims to look beyond surface appearances in order to understand the underlying processes that may account for the phenomena under study [26].

## Discussion

This paper presents a design of a qualitative study to be conducted on service user engagement, the implementation, and delivery of a social prescribing pilot across the four clusters in Luton. The methods of data collection and analysis will enable a thorough study of barriers and facilitators to the implementation and delivery of the pilot, as well as factors affecting service user engagement and disengagement, from various stakeholder perspectives. The critical realist philosophy makes it possible to go beyond lived experiences of interviewees, by exploring possible relationships between contextual factors and the participants’ experiences, views, and behaviour.

It is clear from the literature that the majority of social prescribing interventions in the United Kingdom were not evaluated or limited by poor design and reporting [11,15,16]. Despite the need for a robust evidence base for the emerging concept of social prescribing, most studies that were evaluated poorly report the methodologies, which leaves the quality of the research studies questionable [8,43]. Additionally, most available evaluation reports on social prescribing made reference to factors that facilitate or hinder implementation and delivery, but none looked specifically at these factors. Despite available evidence that patient-level factors predict the implementation and delivery of health interventions [44,45], to the best of the researchers knowledge, there is no study that aimed to understand why primary care patients engage, or do not engage, with social prescribing. Findings of this study aim to fill this existing knowledge gap and may serve as a useful resource to improve the implementation and delivery of future social prescribing interventions.

There are also some limitations to this study, which need to be taken into consideration. For this study, service users, i.e. primary care patients who were referred to the social prescription programme, were interviewed only. There is no data on primary care patients who refused to be referred to the social prescription programme in Luton when a GP suggested a referral during a routine consultation. Therefore this group is not included in the study, and factors that affect the uptake of primary care patients who agree to be referred to the programme is explored exclusively. Future research, including primary care patients who refused to be referred to a SP intervention, is required to understand the barriers to the user uptake of social prescribing programmes.

## Conclusion

The proposed methodology allows a robust qualitative assessment of factors hindering and facilitating the implementation and delivery of social prescribing interventions, as well as factors affecting the engagement, and non-engagement, of service users. Findings of this research can contribute to strengthen the evidence base for social prescription, inform policy and practice, and inform future research in this field.
